# Estimating regional prevalence of chronic hepatitis C with a capture-recapture analysis

**DOI:** 10.1186/s12879-021-06324-z

**Published:** 2021-07-03

**Authors:** Patricia A. M. Kracht, Joop E. Arends, Andy I. M. Hoepelman, Mirjam E. E. Kretzschmar

**Affiliations:** 1 Department of Infectious Diseases, University Medical Center Utrecht, PO BOX 85500, 3508 Utrecht, GA The Netherlands; 2 Julius Center for Health Sciences and Primary Care, University Medical Center Utrecht, Utrecht University, Utrecht, The Netherlands; 3 Center for Infectious Disease Control, National Institute for Public Health and the Environment, Bilthoven, The Netherlands

**Keywords:** Hepatitis C virus, REACH, micro-elimination, Capture-recapture

## Abstract

**Background:**

The hepatitis C virus (HCV) infection is a candidate disease for micro-elimination. Accurate baseline HCV prevalence estimation is essential to monitor progress to micro-elimination but can be methodologically challenging in low-endemic regions like the Netherlands due to lack of disaggregated data by age or risk-groups on the number of chronic HCV patients (i.e. HCV RNA positive). This study estimates the number of patients that has had a chronic HCV infection (ever-chronic) in the Utrecht region of the Netherlands.

**Methods:**

In the Utrecht province in the Netherlands, positive HCV tests from the period 2001–2015 from one diagnostic center and four hospital laboratories were collected. A two-source capture-recapture method was used to analyze the overlap between the two registries (with 92% HCV RNA and 8% HCV immunoblot confirmed infections) to obtain the number of ever-chronic HCV infections in the Utrecht region. The Utrecht region was defined as an area with a 25 km radius from the Utrecht city center. The current viremic HCV prevalence was calculated by taking into account the proportion of cured and deceased HCV patients from a local HCV retrieval (REACH) project.

**Results:**

The estimated number of ever-chronic HCV patients was 1245 (95% CI 1164–1326) and would indicate a prevalence of 0.10 (95% CI 0.09–0.10) in the Utrecht region. This is 30% (95% CI 21–38%) more than the number of known HCV patients in the records. The ever-chronic HCV prevalence was highest in the 1960–1969 age cohort (0.16; 95% CI 0.14–0.18). Since 50% of the HCV patients were cured or deceased in the REACH-project, the number of current viremic HCV patients was estimated at 623 individuals in the Utrecht region (prevalence 0.05%).

**Conclusion:**

The results of this study suggest a low ever-chronic and current HCV prevalence in the Utrecht area in the Netherlands, but other studies need to confirm this.

**Supplementary Information:**

The online version contains supplementary material available at 10.1186/s12879-021-06324-z.

## Background

Estimates based on modelling suggest that 71 million individuals are chronically infected with the hepatitis C virus (HCV) worldwide and consequently are at risk for developing the associated long-term complications such as cirrhosis and hepatocellular carcinoma [1]. Over the past decade, potent direct-acting antivirals (DAAs) have transformed HCV into a candidate infectious disease for global elimination, and to this end the WHO has set out ambitious service coverage targets for eliminating HCV as a public health threat by the year 2030 [2]. The micro-elimination method as a bottom-up approach has been successfully adopted over the recent years [3]. Micro-elimination projects pursue elimination goals with efficient and tailored interventions on a small scale, e.g. in distinct local regions or in specific risk-groups such as persons who inject drugs (PWID) or migrants [4]. Evaluation and monitoring of micro-elimination initiatives requires accurate baseline and follow-up epidemiological data on HCV. Anti-HCV antibodies (anti-HCV) are used to determine seroprevalence of HCV in serosurveys but information on the HCV-RNA positive fraction is also necessary to quantify the viremic population that is still in need of treatment. Obtaining reliable HCV prevalence estimates can be methodologically challenging in low endemic regions like the Netherlands. For instance, serosurveys on HCV often suffer from lack of data disaggregated by age or risk-groups, leading to a high uncertainty in these subgroup estimates [5]. The estimation accuracy of the viremic HCV population can drop even further due to a low or absent HCV RNA positive fraction in (disaggregated) serosurvey data. The Workbook method, which was developed by the WHO for HIV prevalence estimation, aims to overcome this issue and uses risk-group based data to characterize low-level concentrated epidemics such as HCV and HIV [6]. Identifying subgroups at risk however can be challenging, and consequently this approach is still affected by limited data among high-risk groups. A capture-recapture technique can be used to make inference about the total population size by studying the overlap between different data sources [7–9]. This method has been employed in a wide range of settings including laboratory and patient data registries [10, 11] but has not been employed to estimate HCV prevalence in the Netherlands.

In the Utrecht province in the Netherlands, positive HCV test results (i.e. either an anti-HCV or a HCV-RNA PCR) from the period 2001–2015 were extracted from the Laboratory Information System (LIS) of all four regional hospitals and one diagnostic center for general practitioners (GPs) as part of a regional micro-elimination project (REACH) that aimed to identify and trace untreated HCV patients [3]. The current study aimed to estimate the number of individuals in the Utrecht region that have ever had a chronic HCV infection (i.e. the ever-chronic HCV prevalence), which does not take account the proportion of cured or deceased patients and may serve as a baseline indicator for future (micro-)elimination efforts in the Netherlands. The ever-chronic HCV prevalence is estimated by studying the overlap in HCV test results between the hospital and GP diagnostic center with a two-source capture-recapture analysis. In addition, we analyzed the contribution of specific age-cohorts and risk-groups to the HCV burden of disease with additional capture-recapture analyses. Finally, the REACH-project generated information about the proportion of cured and deceased HCV patients, which was used to estimate the size of the current viremic HCV population. Based on the clinical experience of infectious diseases specialists at the University Medical Center Utrecht, we hypothesized that the viremic HCV population would be substantially lower than the 0.16% estimation of the most recent benchmark study on HCV prevalence in the Netherlands [6].

## Methods

### REACH-project

The REACH-project aimed to trace previously diagnosed but untreated HCV patients by extracting positive HCV test-results (i.e. either an anti-HCV test or a HCV-RNA PCR test) from the LIS from all four regional hospitals and one diagnostic center for GPs in the Utrecht region in the Netherlands as published before [3]. Positive HCV test results were obtained from the period 2001–2015 from the hospitals’ LIS. The GPs’ LIS only contained HCV test results from the period 2006–2015 due to a change in the type of LIS that was used in the diagnostic center. The positive HCV diagnostics were linked to clinical records that were subsequently screened by the author (P.K.) to identify those HCV patients who had not yet been cured. Various patient-characteristics were documented, of which data on age, sex and country of birth were relevant to the current study. Untreated HCV patients were invited for re-evaluation at a nearby hepatitis treatment center, which among others included repeated HCV RNA testing. Before contacting patients, the Basic Registry of Persons was consulted based on the individual’s citizen service number to obtain up-to-date contact information, which also included the postal code. This study has been approved by the institutional review board of the University Medical Center Utrecht.

### Capture-recapture

The capture-recapture method originates from the field of ecology where it was often adopted to estimate an animal population size. First, a portion of a population is captured, marked and released. Later on, another sample is extracted from the population and the number of marked individuals in this second sample is counted. If the population mixes homogeneously and every individual is captured with the same probability, the proportion of marked individuals in the second sample is an estimate for the proportion of marked individuals in the entire population. Estimation of the population size can then be obtained by dividing the number of marked individuals by the fraction of marked individuals in the second sample. This approach is often referred to as the Lincoln-Petersen method, named after those who popularized its use [7, 8]. The capture-recapture method has also been adopted in epidemiology to assess the completeness of disease registries [11], and it can be applied to a variety of data sources including health insurance claims, medical prescriptions, but also laboratory and hospital records as was the case in the current study. There are several assumptions to this method, which include: 1) the population is closed (no birth, death or migration); 2) all individuals have the same chance of ‘being caught’ in the second sample; 3) tagging of individuals does not affect their catchability and 4) data sources have to be independent of one another.

**Fig. 1:**
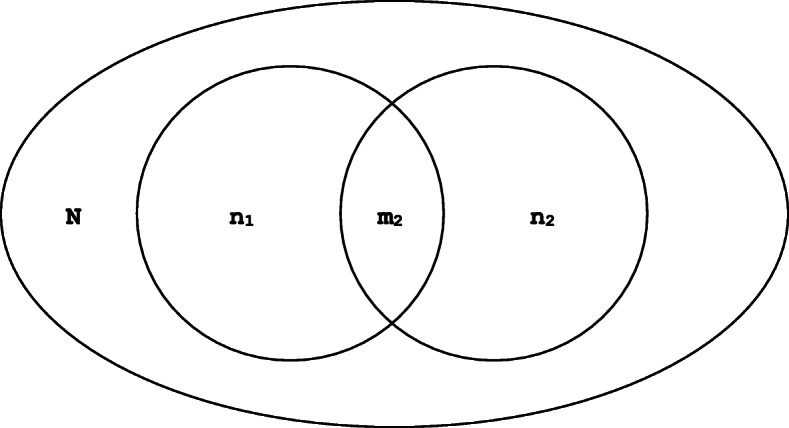
Lincoln-Petersen with Chapman modification. *N* = estimated number of cases in the population; n_1_ = number of cases in first sample; n_2_ = number of cases ‘captured’ in second sample; m_2_ = number of cases that were ‘recaptured’ in second sample; Var = variance; 95CI = 95% confidence interval [7–9]

The modified Lincoln-Petersen estimator with Chapman adjustment was used in this study to estimate the prevalence of chronic HCV in Utrecht with a 95% confidence interval (CI) by means of a two-source capture-recapture analysis (Fig. 1). The two data-sources involved aggregated lists of all positive anti-HCV and HCV RNA tests from: 1) the four regional hospitals’ LIS and 2) the LIS of the diagnostic center for GPs in the Utrecht province. An estimated/recorded population size ratio with 95% CI was calculated by dividing the estimated number of chronic HCV patients obtained from the capture-recapture analysis by the number of originally recorded chronic HCV patients. Since the HCV test results from the GPs LIS could not be acquired from the years 2001 until 2005, there was no overlap to be studied in this period. To overcome this issue, the population size ratio that was acquired by the capture-recapture analysis for the period 2006–2015 was used to estimate the number of patients that have had a chronic HCV infection (*ever-chronic*) in the entire study period (2001–2015). The HCV prevalence in Utrecht was calculated by dividing the result of the capture-recapture analysis by the total population size of the Utrecht *province* on the 1st of January 2016 (Statistics Netherlands - CBS). Finally, the REACH-project generated information about the proportion of cured and deceased HCV patients (=50%) [3]. This proportion was subtracted from the estimated ever-chronic HCV infections to generate an estimation of the current *viremic* population size.

All cleared or cured acute HCV infections and false positive test results were excluded from the analysis through screening of the patient clinical records during the REACH project. The majority of the identified HCV infections (95%) were HCV RNA confirmed and the remaining 5% only had a positive anti-HCV which can indicate both past as well as ongoing HCV infection. Since the latter was a relatively small proportion and progression from acute to chronic HCV occurs in ~ 75% [12, 13] all patients with a positive anti-HCV test, but for whom the HCV RNA test result was lacking, were assumed to have a chronic HCV infection for the purpose of this study. Since the primary outcome of the capture-recapture analysis was the number of ever-chronic HCV infected in the Utrecht region, we initially ignored whether patients had deceased or had already been cured.

$$ N=\left[\begin{array}{l}\left({n}_1+ 1\right)\left({n}_2+ 1\right)\\ {}\kern0.96em \left({m}_2+ 1\right)\end{array}\right]\kern0.5em -1 $$

$$ Var=\left[\begin{array}{l}\left({n}_1+1\right)\left({n}_2+1\right)\left({n}_1-{m}_2\right)\left({n}_2-{m}_2\right)\\ {}\kern2.04em {\left({m}_2+1\right)}^2\left({m}_2+2\right)\end{array}\right] $$

$$ 95 CI=N\pm Z\sqrt{\mathit{\operatorname{var}}(N)} $$

### Utrecht region

The Utrecht province is the smallest province of the Netherlands as its borders demarcate approximately 1400 km^2^, but also one of the most densely populated with approximately 1274 million inhabitants in 2016. The home residencies of the HCV patients in our study sample were located throughout the Netherlands, but only patients living in the Utrecht region were included. For the purpose of the capture-recapture analysis, the Utrecht region was set as the area within a 25-km radius from the Utrecht city center as this most approximated the actual Utrecht province borders, excluding any large cities from neighboring provinces but still including the most important cities from hospitals participating in REACH (including the most distant city Amersfoort of which the outer border is at a linear distance of 25 km from Utrecht) (Fig. 3b). The 25-km radius area spans a total of 1963 km^2^. The individuals’ (last available) residency location was based on their six digit postal code. Patients for whom no information about their current or last available residency was available were excluded from the capture-recapture analysis. The postal codes were translated into geographic coordinates in order to select all HCV patients living within 25-km radius from Utrecht.

### Stratified analysis

The capture-recapture analysis was performed on different HCV patient groups based on the distance of their residency from the Utrecht city center with incremental steps of 10 km (i.e. 10 to a maximum of > 120 km distance from Utrecht). In this manner, the patient subgroups gradually increased in size until the entire study sample was included. For each distance, the estimated/recorded population size ratio with 95% CI was also calculated. In addition, we estimated the number of ever-chronic HCV infected in five specific age cohorts (< 1950; 1950–1959; 1960–1969; 1970–1979; ≥1980) and risk-groups (migrants vs. non-migrants) in the Utrecht region with stratified capture-recapture analyses in these groups. The ever-chronic HCV prevalence in each age cohort in Utrecht was calculated by dividing the result of the stratified capture-recapture analysis by the total population size of each age cohort in the Utrecht *province* on the 1st of January 2016 (Statistics Netherlands - CBS).

The geographical patient selection was done in R studio version 1.1.463 and the “FSA” and “FSAdata” packages were used for the capture-recapture analysis.

## Results

### Patients

**Fig. 2:**
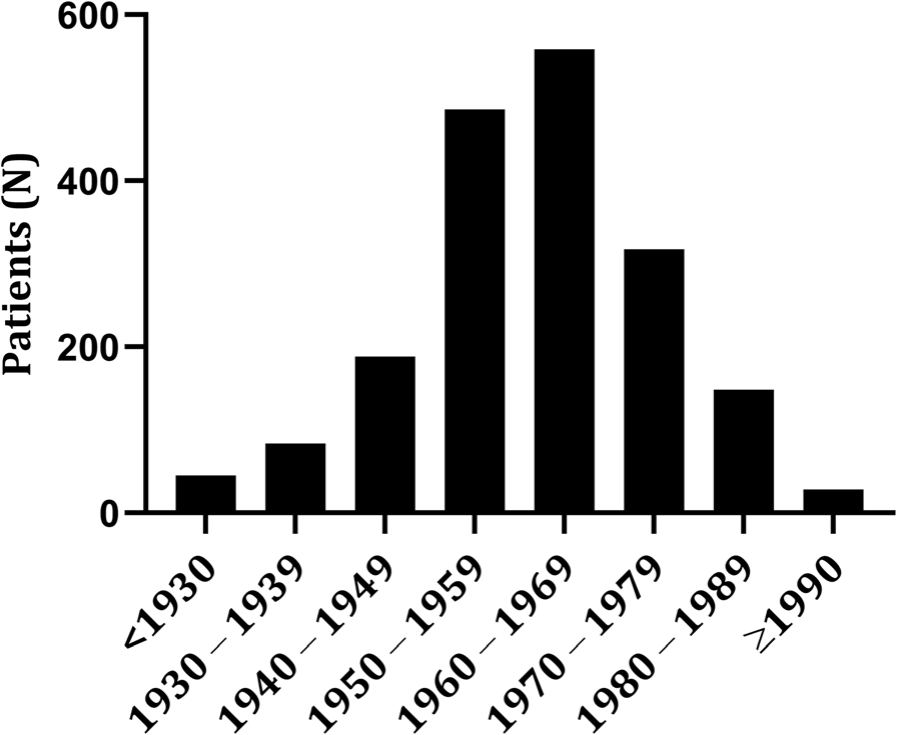
Birth decade of HCV patients who were tested in Utrecht between 2001 and 2015

From the generated laboratory list of positive HCV tests in the Utrecht province, 1853 individual HCV patients could be identified and for 1738 of them, their (last) postal codes were known. Basic patient characteristics are described in Table 1. Patients were predominantly male (74%) and born in the Netherlands (55%), while 45% had a migration background, and of 10%, the country of birth was unknown. More than half (56%) of the HCV patients were born between 1950 until 1969 (Fig. 2). Those HCV patients without a known residency location were more often female (36% vs 25%, p 0.013) and more frequently had an unknown country of birth (50% vs 7%, *p*, < 0.001) compared to those with a registered address. There were no significant differences in the decade of birth between those with and without a known residency location.

**Table 1:** Patient characteristics of HCV patients tested between 2001 and 2015 in Utrecht

	HCV RNA confirmed^**a**^ ***N*** = 1701	HCV immunoblot ***N*** = 152	Total ***N*** = 1853
Gender, n (%)			
Male	1247 (73)	95 (62.5)	1342 (74)
Female	419 (25)	54 (35.5)	473 (26)
Unknown	35 (2)	3 (2)	38 (3)
Country of birth, n (%)			
Netherlands	952 (56)	78 (51)	1030 (55)
Other	596 (35)	49 (32)	645 (45)
Unknown	153 (9)	25 (17)	178 (10)
Residency location, n (%)			
Utrecht region	942 (55)	74 (48)	1016 (55)
Outside Utrecht	671 (40)	51 (34)	722 (39)
Unknown	88 (5)	27 (18)	115 (6)

^a^acute HCV infections had been excluded through screening of the clinical records

### Capture-recapture analysis

**Fig. 3:**
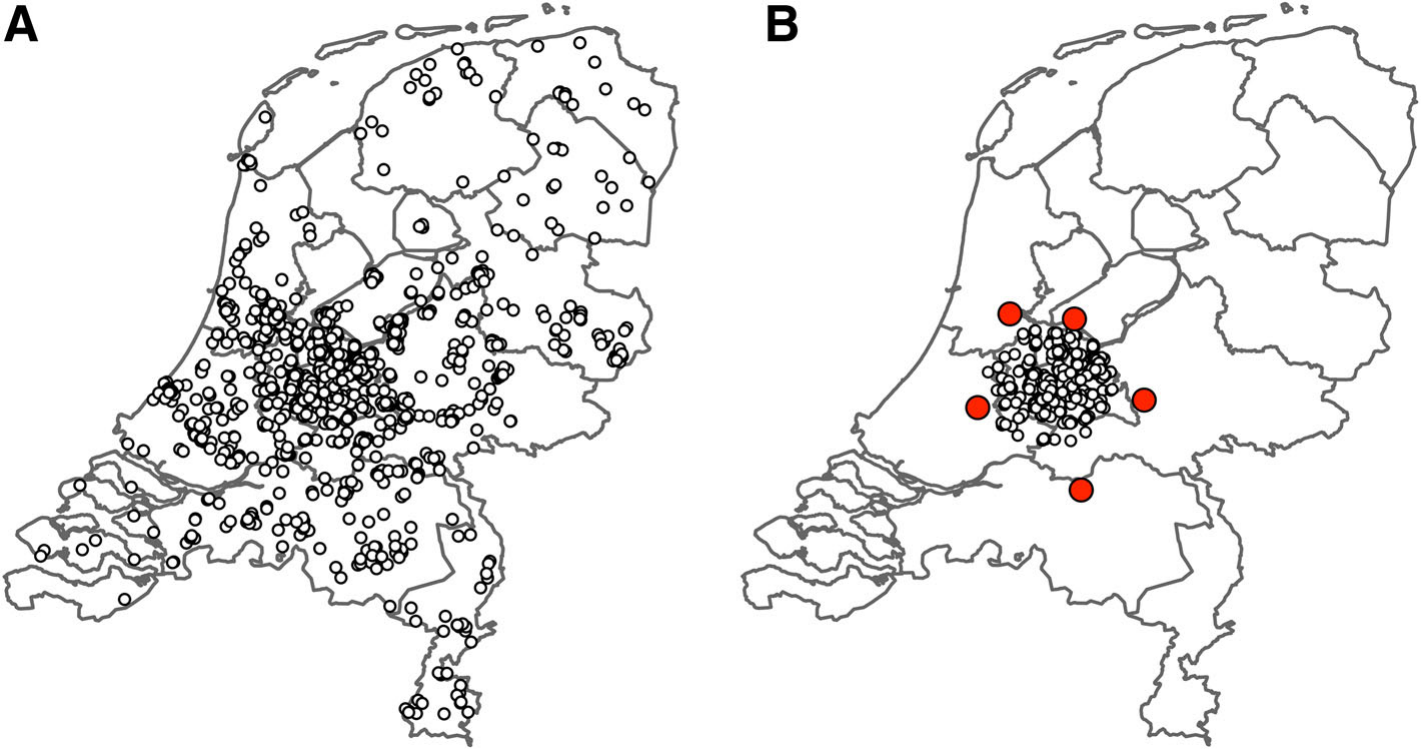
HCV patients who were tested in the Utrecht region between 2001 and 2015. **A** Last known residency of all HCV patients who were tested in Utrecht between 2001 and 2015; **B** Selection of HCV patients who were tested in Utrecht between 2001 and 2015 and of whom the last known residency lays within a 25-km radius from the Utrecht city center. The red dots indicate the five nearest cities not pertaining to the Utrecht province, which were therefore not included in the HCV patient selection: Almere, Amsterdam, Ede, Gouda, ‘s Hertogenbosch. Figure created with R studio version 1.1.463 using the GADMTools package (© 2018 GADM – freely available for academic use)

The geographical location of home addresses of all 1738 known HCV patients was mapped for all recorded HCV patients (Fig. 3a), as well as for those who lived in the Utrecht region (Fig. 3b) for the period 2001–2015. A total of 1016 (55%) HCV patients lived in the Utrecht region. The capture-recapture analysis over the period 2006–2015 resulted in an estimated-to-recorded population size ratio of 1.3 (95% CI 1.21–1.38) in the Utrecht region. This resulted in 1245 (95% CI 1164–1326) estimated ever-chronic HCV infected patients in the Utrecht region. With 1274 million inhabitants, these results indicate an ever-viremic HCV prevalence of 0.10% (95% CI 0.09–0.10). Taking into account the proportion of 50% that already had been cured and/or had deceased in the REACH-project, the number of ever-chronic HCV infections would decrease by 50% to generate the estimated 623 current chronic HCV infections in the Utrecht region. These results would indicate a current viremic HCV prevalence of 0.05% in the Utrecht region.

### Results from stratified analysis

The estimated-to-recorded population size ratios for different patients groups that were selected based on a specified distance of their residency from the Utrecht city center (with incremental steps of 10 km) are depicted in Supplementary Figure 1A. The results of the stratified capture recapture analysis are depicted in Supplementary Figure 1B. The estimated-to-recorded population size ratio gradually increased from 1.1 (95% CI 1.05–1.15) in the10-km radius area to 1.6 (95% 1.47–1.69) for all the recorded patients.

**Fig. 4:**
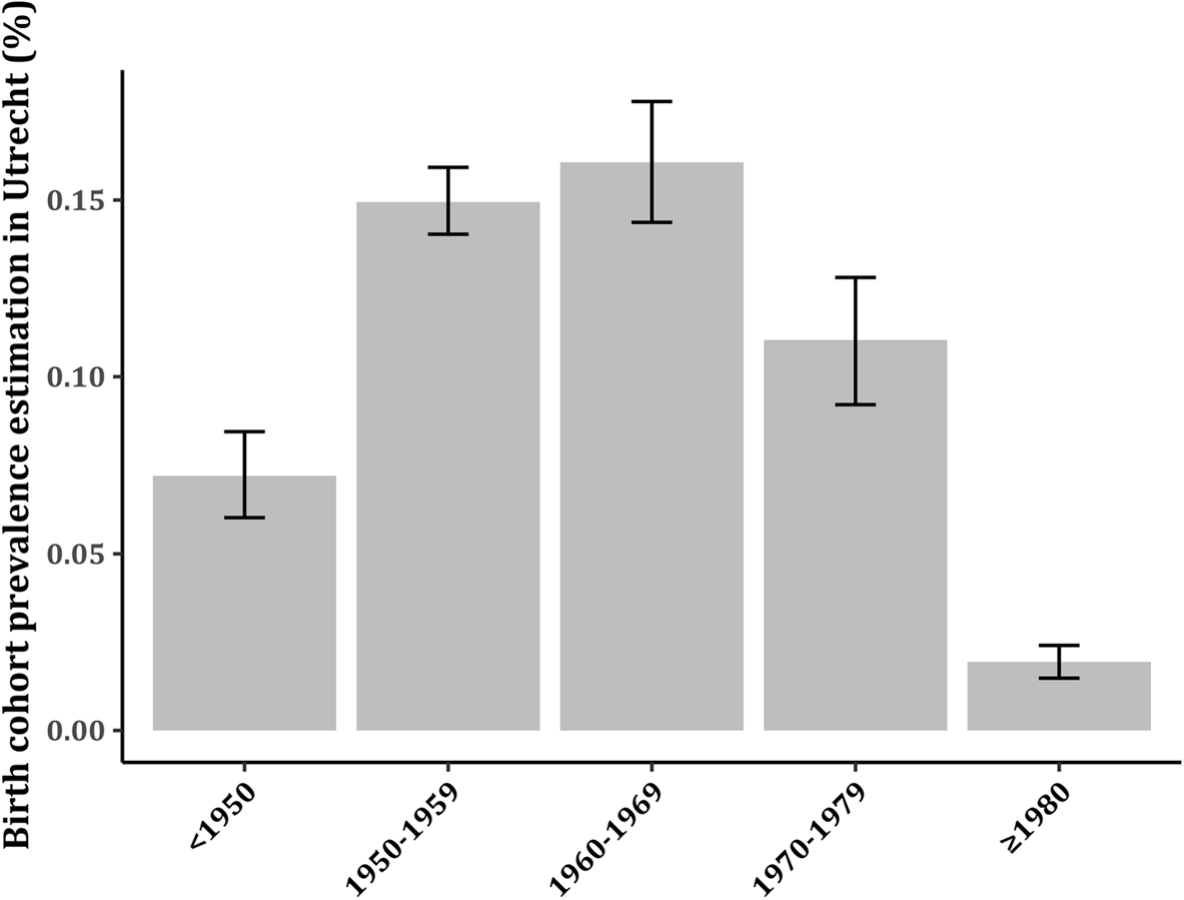
The estimated ever-chronic HCV prevalence per age cohort within the Utrecht region. Ratio of the number of estimated HCV population per age cohort in Utrecht versus the total population size of each age cohort in the Utrecht *province* on the 1st of January 2016 (Statistics Netherlands - CBS)

The estimated-to-recorded population size ratio for five different age cohorts varied between 1.1–1.6 with overlapping confidence intervals. The estimated ever-chronic HCV prevalence in each age cohort is depicted in Fig. 4. For the migrant subgroup, the estimated HCV population size in the Utrecht region consisted of 374 (95% CI 313–450) ever-chronic HCV infected, which constitutes a 25% (95% CI 5–51%) increase of the recorded HCV population. In contrast, the estimated HCV population size of the Dutch natives in the Utrecht region amounts to 528 (95% CI 440–640) ever-chronic HCV infected, corresponding to a 31% (95% CI 9–59%) increase compared to the recorded Dutch born HCV population in Utrecht.

## Discussion

Population size estimation of the population of chronically HCV infected persons in a low prevalence country, where HCV prevalence is concentrated in risk groups, can be methodologically challenging. This study aimed to estimate the ever-chronic HCV prevalence in the Utrecht region by means of a two-source capture-recapture analysis on two extensive lists of positive HCV tests performed between 2001 and 2015 in one diagnostic center for GPs and four hospital laboratories. The number of estimated ever-chronic HCV infected patients in the Utrecht region was 1245 (95% CI 1164–1326), indicating a local prevalence of 0.10 (95% CI 0.09–0.10).

The most recent benchmark study on HCV prevalence in the Netherlands, adopting the Workbook method, reported a slightly higher nationwide ever-chronic HCV prevalence of 0.16% (low 0.06%, high 0.27%) compared to our study, but the confidence intervals overlap [6]. The input data of the Workbook analysis consisted of a combination of heterogeneous studies of which some but not all reported on the HCV RNA positive fraction and also included data from modeling studies. The most recent national cross-sectional serosurvey reported a HCV seroprevalence of 0.3%, but probably does not reflect the current HCV prevalence as it was conducted in 2007. The results of our capture-recapture analysis are based on extensive recent data on HCV tests and suggest a somewhat lower prevalence than the aforementioned HCV prevalence estimates. Our current viremic HCV prevalence estimation, that takes into account cured and deceased patients, is even lower with 0.05%. These findings are supported by a more recent large birth cohort in the south of the Netherlands that tested 3434 individuals in 2014/2015 and found a 0.20% (95% CI, 0.08–0.42%) HCV seroprevalence, but detected no viremic HCV infections [14].

The current capture-recapture approach might, however, have underestimated the total number of HCV infected individuals, since the undiagnosed HCV patients in the population were not sampled. In contrast, between 0 and 8% of the included HCV infections may actually have been cleared or cured HCV infections since they were only HCV immunoblot confirmed. It is probable that this has lead to overestimation of the ever-chronic HCV prevalence. In addition, a closed population was assumed although birth, death and migration did occur. This assumption would probably not have greatly affected the number of ever-chronic HCV infected, which was the main aim of our study. Moreover, the death rate was accounted for by taking the proportion of deceased HCV patients in the REACH-project as a proxy. Also, birth is presumed to have a low contribution to the current chronic infections due to the limited perinatal transmission of HCV (4–10%) in women and because the predominantly male HCV population does not contribute to perinatal transmission [15]. With regard to migration, 7.3% of the recorded HCV patients had been tested in an asylum center, and it is not known what proportion was granted asylum and stayed in the Netherlands and how many moved back to their country of origin. Any bias introduced through this, most likely would have led to overestimation of the ever-chronic HCV prevalence in the Utrecht region. The assumption of independence between the two data sources may be debated since HCV patients diagnosed at the GP are generally referred to a nearby hepatitis treatment center in the Netherlands. For this reason, HCV patients might be registered in both lists more often than would be expected with a random selection. In addition, our results demonstrate that the chance to be included in both databases among others depends on the distance of the home address from the Utrecht city center (Supplementary Figure 1A). A stratified analysis, as performed in the current study, is one of the possible solutions to this issue. When more than two data-sources are available, a log-linear or logit model can be used to model dependence between the different sources by including covariates that may be associated with heterogeneous catchability [16]. Since we only had access to two data sources for the current study, we could not address the question of dependence. Previous studies successfully adopted the capture-recapture method for monitoring and surveillance of infectious diseases. Similarly to our approach, Boender et al. linked the Dutch HIV Monitoring Foundation and the National Registry for Notifiable Diseases to assess annual HIV incidence [17]. In France, a more extensive three source capture-recapture analysis incorporated variables of heterogeneous catchability to estimate the HIV incidence rate in children [18]. A study from Denmark used multiple registries to estimate the national HCV prevalence [11]. In contrast to our analysis, all these studies included national registries, which naturally are preferred to local data sources to prevent the necessity for a (geographical) patient selection such as performed in the current study. Nevertheless, our study demonstrated that a capture-recapture analysis on two data sources can be of additional value to single disease registries for infectious disease prevalence estimation.

To further increase our knowledge on the viremic fHCV population and to monitor micro-elimination progress, the optimal solution would be mandatory registration of both acute and chronic HCV infections. In the Netherlands, mandatory notification of HCV infections to Public Health Services for the purpose of contact tracing is only required for acute HCV infections and chronic HCV infections of unknown duration at the moment [19]. Privacy regulations thus far have precluded further implementation. Either way, future national HCV registries may still benefit from a capture-recapture analysis to assess is completeness.

## Conclusion

The results of the capture-recapture analysis suggest that the number of ever-chronic HCV infections in the Utrecht region is lower than previously assumed. Further studies our needed to confirm these results. To monitor the HCV population with ongoing viremia and the progress to micro-elimination, mandatory notification of all HCV infections is essential. A capture-recapture analysis will remain of additional value to assess completeness of HCV registries in the future.

## Supplementary Information

**Additional file 1: Supplementary Figure 1.** Capture-recapture analysis: the estimated/recorded population size ratio and the HCV population size estimation. **A**: The ratio between the estimated and baseline HCV population size was calculated for each distance including for the 95% confidence intervals. **B**: A capture-recapture analysis was performed on different HCV patients selections based on their residency distance from the Utrecht city center with incremental steps of 10-kilometer. HCV population size estimates with 95% confidence intervals are depicted for each distance.

## Data Availability

The datasets analyzed during the current study are available from the corresponding author on reasonable request.

## Abbreviations

HCV: Hepatitis C virus
DAAs: Direct-acting antivirals
PWID: Persons who inject drugs
anti-HCV: anti-HCV antibodies
LIS: Laboratory information system
GPs: General practitioners
CI: Confidence interval
