# Supplementary figures and images for: Estimating regional prevalence of chronic hepatitis C with a capture-recapture analysis
Source: BMC Infect Dis. 2021 Jul 3;21:640. doi: 10.1186/s12879-021-06324-z (PMC8254300; doi:10.1186/s12879-021-06324-z)

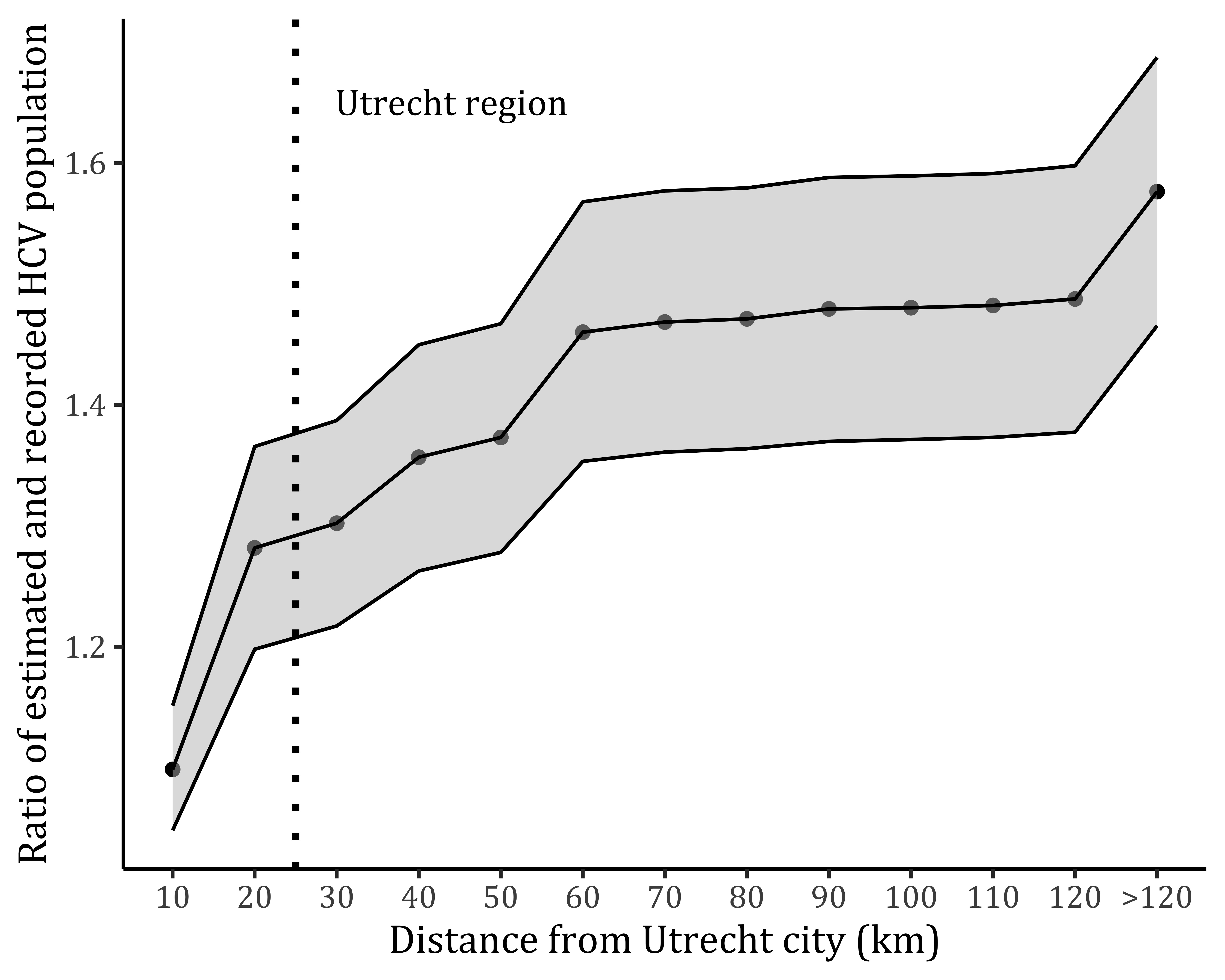

Supplement: Supplementary file 1 — Additional file 1: Supplementary Figure 1. Capture-recapture analysis: the estimated/recorded population size ratio and the HCV population size estimation. A: The ratio between the estimated and baseline HCV population size was calculated for each distance including for the 95% confidence intervals. B: A capture-recapture analysis was performed on different HCV patients selections based on their residency distance from the Utrecht city center with incremental steps of 10-kilometer. HCV population size estimates with 95% confidence intervals are depicted for each distance. [file 12879_2021_6324_MOESM1_ESM.zip › Supplementary Figure 1AR2.jpg]

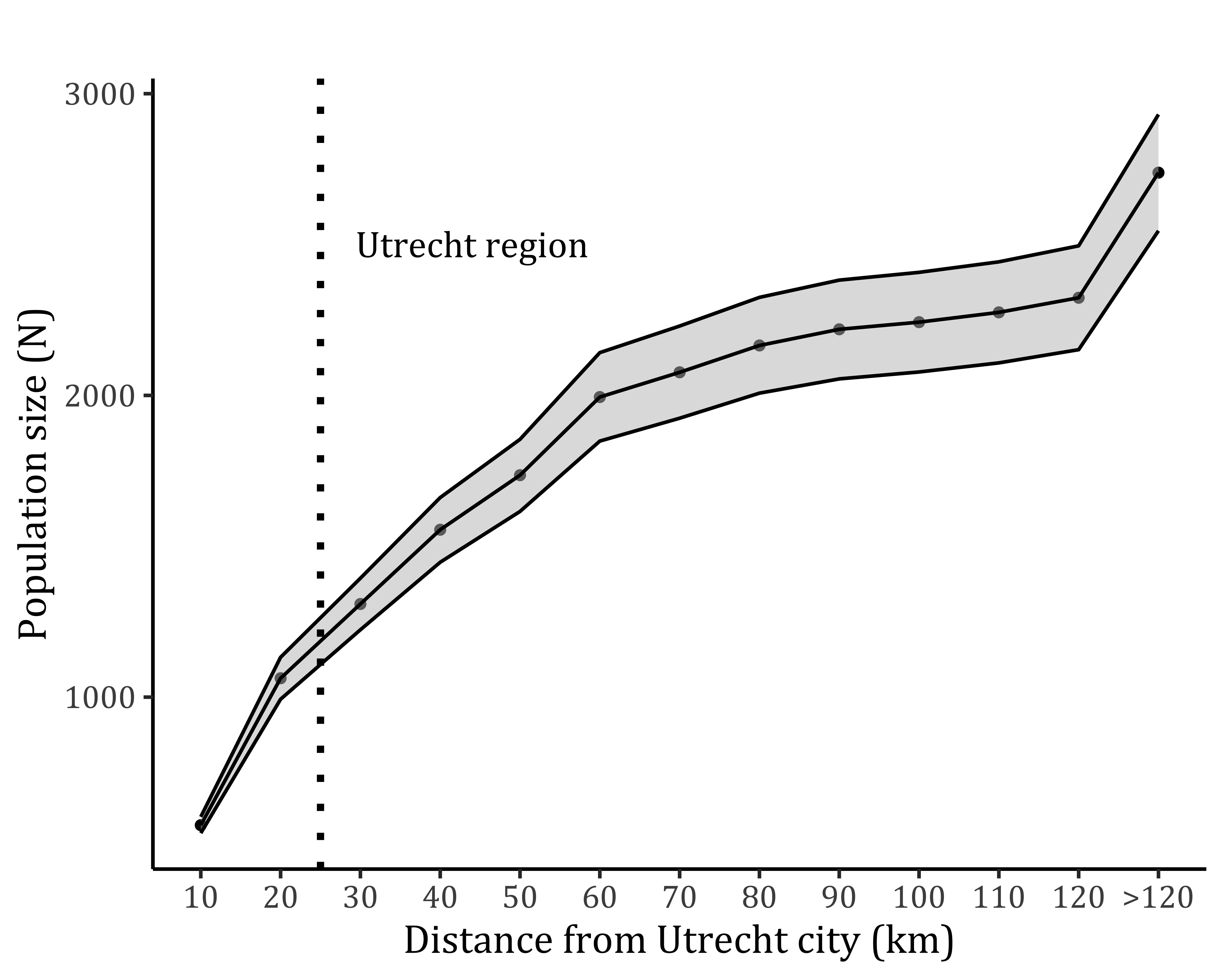

Supplement: Supplementary file 1 — Additional file 1: Supplementary Figure 1. Capture-recapture analysis: the estimated/recorded population size ratio and the HCV population size estimation. A: The ratio between the estimated and baseline HCV population size was calculated for each distance including for the 95% confidence intervals. B: A capture-recapture analysis was performed on different HCV patients selections based on their residency distance from the Utrecht city center with incremental steps of 10-kilometer. HCV population size estimates with 95% confidence intervals are depicted for each distance. [file 12879_2021_6324_MOESM1_ESM.zip › Supplementary Figure 1BR2.jpg]
